# Use of Ozone Therapy in Diabetic Foot Ulcers

**DOI:** 10.3390/jpm13101439

**Published:** 2023-09-27

**Authors:** Álvaro Astasio-Picado, Alba Ángel Babiano, Miriam López-Sánchez, Rocio Ruiz Lozano, Paula Cobos-Moreno, Beatriz Gómez-Martín

**Affiliations:** 1 Physiotherapy, Nursing and Physiology Department, Faculty of Health Sciences, University of Castilla-La Mancha, 45600 Toledo, Spain; albaangelb01@gmail.com; 2 Extremadura Health Service, 10300 Cáceres, Spain; miri-due@hotmail.es (M.L.-S.); rociorulo@gmail.com (R.R.L.); 3 Nursing Department, University of Extremadura, Plasencia (Cáceres), 10600 Plasencia, Spain; pacobosm@unex.es (P.C.-M.); bgm@unex.es (B.G.-M.)

**Keywords:** diabetic foot, ozone therapy, diabetic foot ulcer

## Abstract

Introduction: ozone therapy is a therapy composed of ozone. This gas is in the atmosphere with various general effects: direct disinfectant and trophic effects and a systemic antibacterial and antiviral effect. This gas also improves blood circulation, makes glucose metabolism more effective, improves erythrocyte metabolism, and improves fatty acid metabolism. Objective: Provide evidence of the effectiveness of ozone therapy in wounds of patients with diabetic foot. Analyze the effectiveness of ozone therapy compared to other treatments to achieve good wound healing in patients with diabetic foot. To study the benefits of the use of ozone therapy in ulcers of patients. Analyze the management of ozone therapy and other treatments to achieve healing of ulcers in patients. Methodology: A bibliographic review focused on articles published between November 2014 and June 2023 was carried out. The following databases were consulted: Pubmed (Medline), Dialnet, Google Scholar, Web of Science (WOS), Scielo, and Scopus. Results: After applying the article selection criteria and evaluating the quality of the methodology, a total of 17 articles were obtained. The results affirm ozone therapy as promising for the treatment of wounds in patients with diabetic foot. Conclusions: the evidence has been able to determine that ozone therapy is adequate for the treatment of diabetic foot ulcers. In addition, the therapy has been shown to be effective, safe, and beneficial, with few adverse effects for the treatment of diabetic foot ulcers.

## 1. Introduction

Diabetic foot (PD) is a chronic complication of diabetes mellitus (DM), whose factors are ischemia, infection, poor metabolic control, and neuropathy. Ulcers, gangrene, and amputations appear in the lower extremities of patients with an at-risk foot. This can lead to partial or permanent disability in the patient [1]. Patients with diabetic foot should be admitted to specific units with strategic, permanent, and multidisciplinary care plans, which include treatment and prevention [1]. The diabetic foot must be approached from a multidisciplinary point of view since this problem is highly prevalent, and its complications require the participation of various professionals, such as nursing, orthopedics, podiatry, and medicine [1,2].

### 1.1. Concept, Diagnosis, and Classification of the Diabetic Foot

Diabetic foot is a chronic complication of diabetes mellitus, whose etiology is multifactorial. Its main factors are infection, ischemia, neuropathy, and poor metabolic control. Ulcers, gangrene, and amputations in the lower limbs are frequent clinical characteristics which can cause the patient to be partially or permanently disabled [1,2]. 

It is important to make a correct diagnosis in order to be able to detect the signs and symptoms and to be able to prevent disability and limb loss [2]. To make a correct diagnosis, the anamnesis, physical, neurological, and vascular examination and complementary tests must be evaluated [3]. 

The scales most used to classify the diabetic foot are the Wagner scale, the UT scale (University of Texas), and the scale of the international PEDIS group. The most accepted, known, and simple scale in the world is the Wagner scale. The UT scale classifies the depth of the wound, infection, and peripheral arterial occlusive disease [4]. These categories must be combined with the depth of the ulcer following the Wagner classification [1]. The PEDIS classification evaluates five parameters, these being the most important in the investigation of diabetic ulcers. These categories are evaluated independently [5].

### 1.2. Diabetic Foot Treatment

The diabetic foot ulcer should be cleaned with physiological saline. The use of chlorhexidine, povidone iodine, or hydrogen peroxide is not recommended because it can produce a cytotoxic effect. In the event that the ulcer is necrotic, a debridement is performed in order to eliminate the damaged tissue. Afterwards, the use of synthetic dressings is recommended, which helps to absorb the exudate and keep the ulcer moist, helping its healing [3]. 

After diagnosis, it is initially treated with empirical antibiotic therapy, which can be modified according to the patient’s symptoms and improvement. Before starting treatment, it should be taken into account if the patient presents any resistance to antibiotics and the degree of infection that they present. If the presence of anaerobic or Gram bacteria is anticipated, broad-spectrum antibiotic therapy must be used, with efficacy against anaerobic pathogens [3]. 

Surgical treatment options include debridement and revascularization. Debridement: This treatment is used to achieve adequate healing. Necrotic tissues and bones or fragments that are compromised should be debrided. Normally, lesions appear in the nails, and their removal is necessary in order to drain the purulent secretion. Interdigital and digital lesions can also be observed; depending on their injury, they will need rest, antibiotics, or debridement. The patient’s wound should be constantly observed to avoid complications or future amputations [3]. Revascularization: This treatment is usually used when the patient is unable to perform activities of daily living due to the symptoms they present. The illustration of performing angioplasty is provided by tests such as angiography and Doppler [3].

### 1.3. Ozone Therapy

Ozone is a gas present in the stratosphere whose decomposition rate is high. Ozone is produced by three major sources of energy: electrical discharges, chemical electrolysis, and ultraviolet light radiation [5,6]. 

Ozone presents properties capable of reacting with inorganic and organic substances until the formation of carbon oxides, higher oxides, and water; that is, until its complete oxidation [6]. 

The general effects of this gas are: when applied locally, it has direct disinfectant and trophic effects; thanks to the formation of peroxides, it has a systemic antibacterial and antiviral effect; it increases the deformity of red blood cells, thus improving blood circulation; it favors the delivery of oxygen to the tissues; the metabolism of glucose becomes more effective, improving the metabolism of erythrocytes; finally, thanks to the activation of antioxidant enzymes that are responsible for eliminating peroxides and free radicals, there is an improvement in the metabolism of fatty acids [6]. 

The metabolic effects that can be highlighted are: increased use of glucose at the cellular level, improvement in protein metabolism, and a direct effect on unsaturated lipids, which is responsible for oxidizing it and inducing repair mechanisms at the same time [6]. 

Ozone has various adverse effects; however, it is not a drug, so there are no allergic reactions or interactions with other drugs. When its administration is carried out with high doses, the patient may present a feeling of heaviness. This usually occurs in a few patients, with a short duration and rapid resolution. Rarely, the painful stimulus due to the puncture of the needle can cause a vagal crisis in the patient without the need to use pharmacotherapy to reverse it [6]. 

Patients in whom the use of this therapy will be contraindicated will be patients with a large glucose six phosphate dehydrogenase deficiency since red blood cells may oxidize and cause hemolysis. It should not be performed in patients with thrombocytopenia and hyperthyroidism either. It should also not be performed in those with severe cardiovascular instability, hemorrhagic pictures, or convulsive status [6].

The objective of this study is to analyze the effectiveness of ozone therapy in wounds of patients with diabetic foot, analyze the effectiveness of ozone therapy compared to other treatments to achieve good wound healing in patients with diabetic foot, to study the benefits of the use of ozone therapy in ulcers of patients, and analyze the management of ozone therapy and other treatments to achieve healing of ulcers in patients.

## 2. Materials and Methods

The preparation of this work was carried out through a systematic bibliographic review of the articles found by searching the following databases: Medline/Pubmed, Dialnet, WOS, Scielo, Scopus, and Google Scholar. To determine the best possible scientific evidence, a series of inclusion and exclusion criteria were applied.

### 2.1. Information Sources and Search Strategy

The keywords for this review are: diabetic foot, ozone therapy, and diabetic foot ulcer. To carry out the bibliographic search, different keywords in English were used, such as: “diabetic foot”, “ozone therapy”, “ozone therapy”, “diabetic foot”, “ozone therapy”, and “ulcers”. These were validated by DeCS and MeSH. Once selected, the corresponding Boolean operators were used: AND, as well as the necessary parentheses and quotation marks. The final search string was as follows: ((Diabetic foot) AND (Ozone therapy)). 

### 2.2. Inclusion Criteria and Exclusion Criteria

The criteria that were taken into account for the selection of the relevant studies were the following. Inclusion criteria: the period between 2014 and 2023; article type: article review and article research; field: medicine; English language; sample in the adult population; studies that provide scientific evidence justified by the level of indexing of articles in journals according to the latest certainties. Exclusion criteria: articles prior to 2014; language: not English; studies in which the population included minors; studies that do not provide scientific evidence justified by the level of indexing of articles in journals according to the latest certainties.

### 2.3. Methodological Evaluation of the Data Used

For the methodological evaluation of the individual studies and the detection of possible biases, the evaluation was carried out using the PEDro Evaluation Scale. This scale consists of 11 items, providing one point for each element that is fulfilled. The articles that obtained a score of 9–10 points have excellent quality, those between 6 and 8 points have good quality, those that obtained 4–5 points have intermediate quality, and, finally, those articles that obtained less than 4 points have poor methodological quality article.

The Scottish Intercollegiate Guidelines Network classification was used in the data analysis and assessment of the levels of evidence, which focused on the quantitative analysis of systematic reviews and the reduction in systematic error. Although it took into account the quality of the methodology, it did not assess the scientific or technological reality of the recommendations.

## 3. Results

**Scheme 1:**
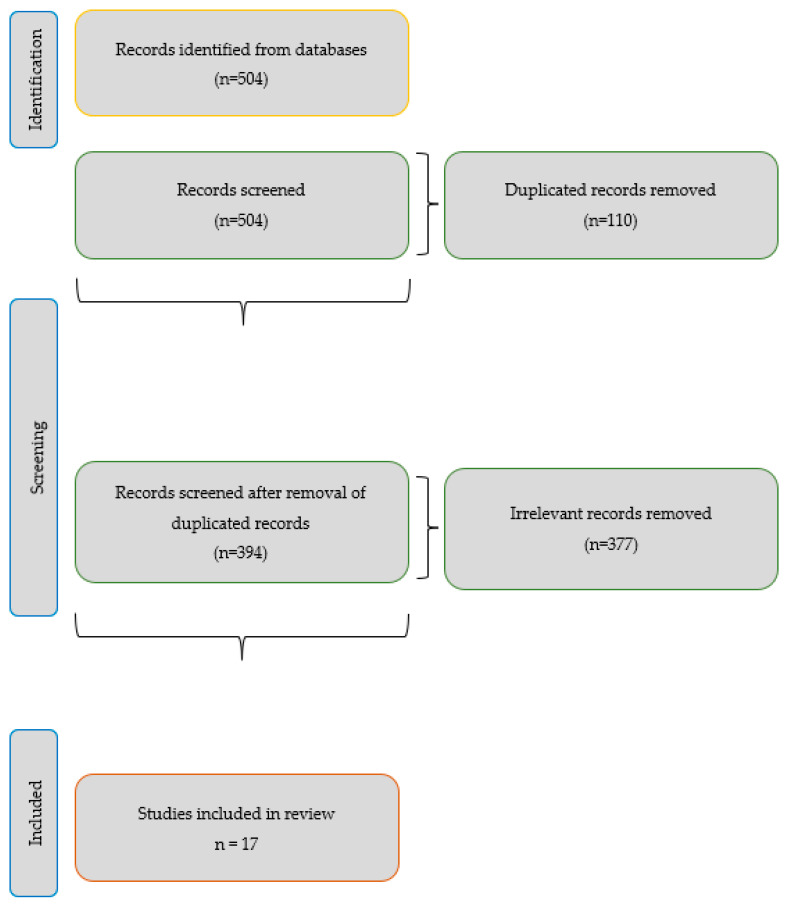
Flow diagram.

The research question was constructed following the PICO format (population/patient, intervention, comparator, and outcomes/outcomes), detailed as P (patients): adult subjects of both sexes; I (intervention): use of ozone therapy in patients with diabetic foot ulcers; C (comparison): traditional treatments for ulcers in patients with diabetic foot; O (results): effectiveness of ozone therapy (Scheme 1).

Below is a table that shows the search strategy used to select the 17 articles selected from the six databases, following the criteria of identified studies, duplicate studies, title, abstract, full text, and valid studies of a definitive nature. The total number of valid articles is summarized in Appendix A.

In the studies by Suchin Dhamnaskar et al., Jing Zhang et al., Jaróslaw Pasek et al., Xiaoxiao Hu et al., and Laura Gheuca Solovastru et al., no change in wound area was observed at the beginning of treatment (11.742% versus 10. 82%), but 21 days after ozone administration, a reduction in the wound area of 18.62% could be seen; with statistical significance, its values were <0.001 and 0.022, respectively (Table 1) [7,8,9,10,11].

In the study by Jaróslaw Pasek et al., a reduction in area was observed, taking into account sex, age, BMI (body mass index), duration of the disease, and location of the ulcer (Table 2) [9].

After the administration of ozone treatment, there was a reduction in the wound area of 38.74%. Fifty-four patients participated in the study; 2 of the participants healed completely (3.7%), 18 of them achieved a reduction greater than 50% of the initial value (33.3%), and the remaining 34 patients observed a smaller reduction of 50% of the initial value (63.0%) [9]. 

In the study by Jaróslaw Pasek et al., they observed a 60% reduction in pain. In 2 of the patients, a complete reduction was achieved (3.7%), in 48 patients, the pain was greater than 50% of the baseline pain (92.5%), and in 4 patients, the pain intensity was reduced but did not exceed 50% of baseline (7.4%) [9]. 

In the articles by Yi-Ting Zhou et al. and Mufarika et al., healing was taken into account, being greater in patients treated with ozone therapy. After 12 months of follow-up of this treatment, the patients who were treated with ozone obtained a healing of 92%, while the rest of the patients treated with standard treatment obtained a healing of 76.19% in the first study. In the second study, the mean value before the test was 42.77 and after the test 20.69 after ozone application [12,13]. In the trial by Morteza Izadi et al., they also discussed the healing time, being 69.44 ± 36,055 days in the ozone group, while in the control group, after 180 days, 25% of the patients did not heal completely [14]. 

In the study by Suchin Dhamnaskar et al., they observed that patients treated with ozone had 20% more granulation tissue than conventionally treated patients [7]. In the study conducted by Myroslav V. Rosul et al., a reduction in the burning sensation in the foot, a constant cooling of the feet, and paresthesia could be observed [15].

Erin Fitzpatrick et al. carried out a systematic review where they reviewed various trials in which different results could be observed, as well as different age ranges and duration of treatment (Table 3) [16]:

Kasmawati Kadir et al. carried out a test where they observed a significant reduction in the number of bacteria in the group treated with ozone (*p* = 0.001), while in the control group, there was no reduction (*p* = 0.06). The number of bacteria was higher before treatment (*p* = 0.334) than after it (*p* = 0.037) [17]. 

Navid Faraji et al. carried out a case report on a patient who underwent several ozone therapy sessions for 30 days. After the sixth session, the deep parts of the wound were closed as the granulation tissue grew rapidly. After one month of treatment, the ulcer had completely closed [18]. 

A systematic review was carried out by Andressa Urbano Machado et al., where they were able to compare various trials and obtain different results (Table 4) [19].

Lima Bomfim et al. carried out a systematic review, where different results were observed in various trials in which they used ozone for the treatment of wounds (Table 5) [20].

Gheuca Solovastru et al. carried out a study where changes in the mean speed of healing could be observed. In the first 7 days, it was reduced by 0.17 cm^2^, 0.14 cm^2^ between days 7 and 14, and 0.04 cm^2^ in the last days of treatment [11]. Aparecida Oliveira Modena et al. carried out a systematic review, which showed several trials with different results in each one (Table 6) [21].

Quing Wen et al. and Svitlana Y. Karatieieva et al. carried out studies where various results could be observed, highlighting an improvement in healing and a decrease in the amputation rate and wound area [22,23]. 

Jing Zhang et al. conducted a study where various changes in growth factors could be seen. They compared the ozone group with the control group. At the beginning of the treatment, they could not observe major differences in vascular endothelial growth factor (VEGF), with 19.95% in the ozone group and 17.93% in the control group. After administration of ozone treatment, the results increased to 34.86% in the ozone group and 26.44% in the control group [8]. 

They also studied transforming growth factor (TGF). When ozone was administered, the values of the ozone group were 4.48%, and those of the control group were 5.17%. After its administration, there was an increase in these values, that of the ozone group being 14.95% and the control group being 10.45% [8]. 

They also looked at the results for platelet-derived growth factor (PDGF) proteins. When ozone treatment was administered, the values were 14.23% in the ozone group and 15.50% in the control group. After its administration, the values were 31.44% in the ozone group and 20.78% in the control group [8].

## 4. Discussion

The studies reviewed in this research paper provide information on ozone therapy in wounds of patients with diabetic foot. 

Several authors discuss the reduction in the amputation rate thanks to the use of ozone therapy. Morteza Izadi et al. determined a decrease in C-reactive protein and in the erythrocyte sedimentation rate; by reducing this, the amputation rate was also reduced [14]. Lima Bomfim et al. demonstrated that ozone was an effective adjuvant therapy, helping to prevent complications in the disease, as well as amputations. In addition, to improve the results they obtained, they wanted to carry out evidence-based protocols for ozone treatment [20]. Quing Wen et al. stated that the quality of the studies they included was poor; despite this, thanks to the use of ozone, both individually and in combination, they were able to observe a reduction in the amputation rate [22]. 

According to Suchin Dhamnaskar et al., the use of ozone accelerated the formation of granulation tissue. After 21 days of treatment administration, wound healing was 27% faster in the ozone group, and signs of inflammation disappeared [7]. Myroslav V. Rosul et al. used ozone both locally and systemically, which improved the appearance of granulation tissue [15]. 

Regarding the reduction in the wound area, Quing Wen et al. observed a decrease in this but did not contemplate an improvement in the proportion of wounds completely healed [22]. Gheuca Solovastru et al. perceived a significant and progressive reduction in the wound, being superior to the control group, which did not improve after the observation period [11]. On the other hand, in the study by Yi-Ting Zhou et al., no adverse effect was observed [12]. Jaróslaw Pasek et al. considered the decrease in area, age, sex, body mass index, duration of the disease, and location of ulcers [9]. 

Regarding hospital stay, Suchin Dhamnaska et al., Lima Bomfim et al., and Myroslav V. Rosul et al. observed a reduction in this in each study. In the study carried out by Myroslav V. Rosul, the hospitalization time of the ozone group was 17.09 days [15]. However, in the study carried out by Suchin Dhamnaska et al., it was 9 days. By reducing hospitalization time, overall cost expenditure was indirectly reduced [7]. Lima Bomfim et al. observed an improvement in wound healing and, as a consequence, a reduction in the stay of patients in hospital [20].

To evaluate the extent of pain, both Jaróslaw Pasek et al. and Xiaoxiao et al. used the visual analogue scale (VAS). To evaluate pain, Jaróslaw Pasek et al. took into account age, sex, body mass index, duration of the disease, and location of the ulcer. In this study, a reduction in pain could be observed, although it was reduced more in some patients than in others [9]. On the other hand, although Xiaoxiao et al. also experienced pain reduction, they admitted that this treatment needs more research since the sample was small and all cases were performed at the same center [10]. Regarding the use of VAS, a statistically significant reduction in pain intensity on the VAS scale was evident in all patients, median of 6 (5–7) points before treatment versus 4.4 (3–7) points after treatment, *p* = 0.000001) [9]. Similarly, treatment duration was significantly shorter and dressing change times and maximum VAS scores were dramatically lower in the combined group than in the VAC group [10].

Ozone therapy hastened the healing process in several studies. According to Morteza Izadi et al., ozone decreases the FBS (fasting blood sugar) level, improving wound healing [14]. In the study by Qing Wen et al., healing in the ozone group was not much higher than in the control group 12 months after treatment, so the quality shown in this study was low [22]. According to Suchin Dhamnaskar et al., the number of ozone sessions administered and healing could have a certain relationship, but more studies are needed to prove it [7]. Jing Zhang et al. commented that diabetic foot ulcer healing was improved after control of platelet-derived growth factors and transforming growth factors [8]. According to Gheuca Solovastru et al., from practice, healing depends on various factors, such as the preparation of the wound bed [11]. Mufarika et al. observed healing acceleration thanks to the use of ozone on wounds compared to patients who were not administered this [13]. 

Navid Faraji et al. carried out a case report after ozone administration, and an acceleration in wound healing was observed, thus improving the patient’s quality of life [18]. On the other hand, although Kasmawati et al. did not perceive changes in wound healing, she was able to see how changes were made in the bacteria presenting it, reducing their colonies [17]. 

Fitzpatrick et al., Urbano Machado et al., and Aparecida Oliveira Modena et al. conducted a systematic review. According to Fitzpatrick et al., thanks to their meta-analysis, they saw that there is good evidence of the use of ozone, in addition to being effective, but more research is needed on the matter [16]. While Aparecida Oliveira et al. studied its short-term efficacy, more studies are needed to determine what the adverse effects are [21]. Urbano Machado et al. perceived several adverse effects; despite this, they concluded that ozone can be used as a complementary treatment [19].

In the same way, in the study carried out by Svitlana Y. Karatieieva, she saw that ozone did not negatively affect the levels of intoxication of the body or homeostasis; in addition, it improved the evolution of the wound in purulent processes [23].

## 5. Conclusions

In relation to the main objective of demonstrating the effectiveness of ozone therapy in wounds of patients with diabetic foot, it was verified that the use of ozone therapy has positive effects on wounds of patients with diabetic foot, accelerating their healing and scarring, in addition to improving the quality of life of the patient. This refers to the objective of analyzing the effectiveness of ozone therapy compared to other treatments to achieve good wound healing in patients with diabetic foot. It has been shown that ozone therapy positively affects the wounds presented by patients with diabetic foot. Its use, compared to other treatments, accelerates wound healing, which leads to a reduction in cost and hospital stay. Despite this, little research has been conducted on ozone therapy, so more research is needed on this therapy. Regarding the objective of studying the benefits of the use of ozone therapy in ulcer patients, great results have been obtained. It was evidenced that it is an effective, safe, and beneficial therapy, and few adverse effects have been observed. The wounds presented better healing, a reduction in their area, a decrease in bacterial colonization, and a reduction in the amputation rate, reducing hospital stay and, consequently, reducing cost, as mentioned above. Finally, the objective is to analyze the management of ozone therapy and other treatments to achieve the healing of ulcers in patients. It was concluded that the use of a standard treatment and ozone therapy simultaneously has beneficial effects, accelerating the complete healing of the wound.

## Figures and Tables

**Table 1:** Reduction in the wound area (Source: own elaboration).

Studies	Wound Area Reduction
Suchin	21 days later, it was reduced by 18.62%
Jing Zhang	20 days later decreased <0.001
Xiaoxiao Hu	Greater reduction 1 week later *p* < 0.05
Laura Gheuca Solovastru	After 7, 14, and 30 days, it was reduced by 34%, 59% and 73%

**Table 2:** Wound reduction (Source: own elaboration).

Sex. Age. BMI. Duration. Wound Location.	Wound Area Reduction
Male vs. female	0.000012 vs. 0.000003
<60 years vs. 60–70 years vs. >70 years	0.00004 vs. 0.000655 vs. 0.000293
Normal weight vs. overweight	0.000006 vs. 0.000006
<5 years vs. >5 years	0.000001 vs. a 0.000132
Left leg vs. right leg	0.000003 vs. a 0.000012

**Table 3:** Comparison of results in a systematic review (Source: own elaboration).

	Marfella	Martínez-Sanchez	Zhang	TurcoC	Borrelli	Wei	Campanati	Solovastru	Wainstein
Age	5 to 70	20 to 80	12 to 60	4 to 25	16.1 to 64.4	48.4	14.3 to 52.5	18 to 30	10.2 to 62.6
Treatment duration	22 weeks	20 days	20 days	10 days	2 days	25 days	12 weeks	30 days	12 weeks
Cicatrization	<TNF-a levels: 225.5–28.2 a 114.8–23.1 ng/L	74.58–0.35%	Changes in size 6.84–0.62 cm^2^	75% total closure	80% total closure	85% total closure	Changes in erythema	25% total closure	81% full closure

**Table 4:** Comparison of results in a systematic review (Source: own elaboration).

Author	Treatment Period	Improvements in the Ozone Group
Hassan	3 months	Increase in the % cure in those treated with ozone.
Wainstein	12 weeks	Higher proportion in total closure of the wound (81% vs. 44%).
Zhang	20 days	Significant wound reduction (*p* < 0.001). Global effective rate of 92%.
Solovąstru	30 days	Wound reduction (*p* < 0.05). Complete closure in 25% of patients.
Teuvov	According to each patient	Reduction in days in bed and microbial bodies. Increases regeneration speed.
Hassanien	20 days	96% healing rate. Increase in VEGF and reduction in antiETAR (*p* < 0.001).
Izadi	180 days or complete closure	100% total closure, decreased healing time (*p* = 0.012), lower % of amputation (*p* < 0.05).
Kadir	21 days	Reduction in the number of bacterial colonies (*p* = 0.037).
Kaymaz	4 weeks	Healing effectiveness rate of 92%. Decrease in size (*p* = 0.027) and pain (*p* < 0.01).
Menendez	3 months	90.5% were completely cured, 9.5 improved their condition.
Lu	4 weeks	There were no differences between the two groups.
Illek	3 months	Disappearance of symptoms after the end of treatment. They appear 10 months later in the ozone group and three months later in the control group.
Zeng	3 days	Bacterial skin restoration (*p* = 0.023).
Campanati	12 weeks	Patients treated with ozone presented less intense hyperpigmentation after healing (*p* = 0.03).
Martusevich	15 days.	No significant differences were observed between both groups.
Curro	7 days	Increase in growth factors (*p* < 0.001).
Aghaei	2 months	Decrease in size, there were no significant differences. Mild post-inflammatory hyperpigmentation in the ozone group after healing.
Ibrahim	Maximum 10 sessions or until full authorization.	Response to treatment of 90.9%. Recurrence rate after 6 months of treatment (5% vs. 15%).

**Table 5:** Comparison of results in a systematic review (Source: own elaboration).

Author	Evaluation Method	Main Results of the Ozone Group
Solovastru	Patients evaluated on days 0, 7, 14, and 30	In the control group, there was 25% complete healing. There was a significant reduction at 7, 14, and 30 days of 34%, 59%, and 73%.
Izadi	Wagner’s classification for wounds and a ruler to measure the wound were used.	There was a complete healing of the wounds in the ozone group, with a mean time of 69.44 days.
Rosul and Patskan	The healing process was determined by the tissue presenting the wound.	At 10.17 days, thanks to the use of ozone, the pain, swelling, and hyperemia observed around the wound were reduced. Granulation was observed at 14.46 days.
Zhou	Patients were evaluated one week after treatment and then evaluated monthly until healing.	Healing was greater in the ozone group; it was 92%, and there was greater recurrence and satisfaction. No adverse effects were observed.
Wainstein	The wound area was measured thanks to a transparent grid. Infections were evaluated by bacterial cultures.	Patients who were treated with ozone had a closure rate of 81%.
Zhang	Patients were evaluated at baseline, on day 11, and on day 20. Width, length, depth, healing, infection, and need for debridement were evaluated.	In the evaluation on day 20, a reduction in the wound was observed, being greater in the ozone group. After the administration of ozone, the wounds had more collagen fibers.
Kadir	On days 0 and 21, bacterial colonies were evaluated.	There was a reduction in colonies in the ozone group. Extensive scarring was also observed in this group.

**Table 6:** Comparison of results in a systematic review (Source: own elaboration).

Author	Number of Sessions	Conclusion
Davatdarova and Kazimov	3–4 times a week.	The use of ozone was effective in the ozone group.
Gloor and Liphardt	Once a day for a week.	Ozone was ineffective for various microorganisms.
Guizhi	3–5 times a week for 2 weeks.	The ozone was effective and efficient.
Jianyun	2 times daily per week.	It reduced the effects of colonization by *S. aureus*.
Gao	2 times daily for 4 weeks.	Ozonated oil is a safe and effective method to use.
Lina	2 times daily for 4 weeks.	Ozonated oil is effective, equivalent to glucocorticoid ointments.
Hassaniem	1 time daily for 20 days.	Rapid healing of the wound.
Nowicka	2 times daily for 10 days.	Increase in joint mobility and temperature, reduction in thickness.
Jian	1 time daily for 5 days.	Decrease in pain in the acute phase and reduction in the duration of the virus.
Wainstein	4 times a week for 4 weeks.	It is beneficial when ozone is combined with conservative treatment.
Martínez-Sánchez	1 h each day for 20 sessions.	It was considered effective and useful as an alternative treatment.
Campanati	1 time daily for 12 weeks.	It was effective.

## Data Availability

Not applicable.

## Abbreviations

QALY	Quality-adjusted life year
DM	Diabetes Mellitus
VAC	Vacuum-assisted Closure Therapy
DeCS	Descriptors in Health Sciences
MeSH	Medical Subject Heading
SJR	Scimago Journal and Country Rank
SIGN	Scottish Intercollegiate Guidelines Network

## Appendix A. Selected Scientific Articles Table

**Table:**

Authors; Year	Type of Study	Patients	Conclusion
**Morteza Izadi. et al. 2019 [14].**	Single-blind randomized clinical trial	200 patients: 100 patients treated with ozone, usual conservative treatment, 100 patients treated with routine care.	Objective: to be able to identify the efficacy and safety of ozone in ulcers of patients with diabetic foot. Conclusion: ozone therapy reduces healing time, allows better healing, reduces C-reactive protein and sedimentation rate, and decreases the amputation rate.
**Kasmawati Kadir. et al. 2020 [17].**	Non-randomized controlled study	27 patients: 14 treated with standard care, bandage, oxygen-ozone therapy, 13 treated with bandage, standard care.	Objective: to identify the effectiveness of the combination of ozone therapy and standard wound treatment in order to reduce bacterial colonies and stimulate wound healing. Conclusion: the combination of ozone therapy at 70 μg/mL and the standard care performed significantly reduces the number of bacteria in wounds, but no significant changes in healing are shown.
**Qing Wen. et al. 2021 [22].**	Systematic review	12 articles were obtained, with a total of 1055 participants in total.	Objective: To evaluate the efficacy and safety of ozone therapy in chronic ulcers. Conclusion: ozone reduces the wound area and decreases the amputation rate in patients with diabetic foot ulcers, but this is not evident in completely healed ulcers.
**Suchin Dhamnaskar. et al. 2021 [7].**	Observational prospective comparative study of cohorts	162 patients: 81 of them were treated with debridement, cleaning, Betadine, and bandage; the other 81 were treated with the previous treatment together with topical ozone.	Objective: to assess the safety and efficacy of topical ozone gas used in diabetic foot ulcers. Conclusion: during an acceleration in the change in exudate, ozone allowed a more rapid creation of granulation tissue, promoted the disappearance of inflammation, reduced microbial colonization, and improved healing of the wound edges. In addition, hospital stay was significantly reduced.
**Tássia Lima Bomfim. et al. 2021 [20].**	Systematic review	44 articles were obtained at the beginning, and after a selection, the final result was 7 articles.	Objective: to summarize the different evidence on the therapeutic use of ozone in people with venous ulcers to promote decision-making on the use of this treatment, improve wound healing, and enhance patient safety. Conclusion: they support the effectiveness of this therapy as an additional treatment for ulcers, it reduces the rate of amputation, infection, and hospitalization, but more studies are needed to prove its efficacy in healing ulcers effectively and safely.
**Jing Zhang. Et al. 2014 [8].**	Randomized controlled prospective clinical study.	50 patients: 25 patients were treated with non-invasive ozone and oxygen treatment together with standard treatment, and the other 25 patients were treated only with standard treatment.	Objective: to observe the effects of ozone therapy on the healing of diabetic foot ulcers and the expressions of vascular endothelial growth factor (VEGF), transforming growth factor (TGF), and platelet-derived growth factors (PDGF) of these after treatment. Conclusion: ozone and oxygen treatment significantly promoted wound healing, and an increase in VEGF, TGF, and PDGF expressions was observed in the ozone group. The effectiveness of this treatment in healing, according to these results, may be due to the increase in endogenous growth factors.
**Erin Fitzpatrick. et al. 2018 [16].**	Systematic review	In the first search, 12 articles were obtained. After the exclusion criteria, 9 articles were obtained.	Objective: to investigate the possibility of ozone therapy as an effective medical procedure for use in chronic wounds. Conclusion: ozone therapy has positive effects on chronic ulcers, but it is necessary to carry out more research to agree on a specific application method to obtain the most appropriate dose for its effectiveness.
**Xiaoxiao Hu. et al. 2019 [10].**	Prospective study	136 patients: 68 patients were treated with VAC + ozone water wash, and 68 patients treated with VAC.	Objective: to find out the efficacy of treatment with vacuum negative pressure and washing with ozone water for diabetic foot ulcers. Conclusion: the use of these two drugs in combination produces an improvement in the recovery of ulcers and reduces the duration of treatment and pain during it.
**Jarosław Pasek. et al. 2022 [9].**	Prospective study	54 patients were treated with local ozone therapy: 25 male patients and 29 female patients.	Objective: to examine the efficacy of local ozone therapy in venous ulcers, taking into account the area of the wound and its pain. Conclusion: the application of local ozone therapy reduces the surface of the wound and the intensity of the pain perceived by the patient.
**Urban Machado. et al., 2022 [19].**	Systematic review	30 trials: 12 trials were withdrawn as they did not meet the inclusion criteria. 18 articles were used to conduct this review.	Objective: to assess clinical trials of the last 10 years on the safety and effectiveness of ozone therapy used in dermatological conditions. Conclusion: ozone therapy is effective, and it could be used as an alternative or complementary treatment in dermatological conditions such as diabetic foot ulcers, but more research should be carried out on its risk–benefit ratio.
**Aparecida Oliveira. et al. 2020 [21].**	Systematic review	326 articles: after the exclusion criteria, the final result was 17 articles.	Objective: to determine the safety and efficacy of ozone therapy in various dermatological conditions. Conclusion: in the short term, ozone therapy is effective, especially in ulcers and scars, but more research is still necessary to observe in the long term what are the adverse effects that it can present.
**Svitlana Y. Karatieva. et al. 2021 [23].**	Prospective study	122 patients participated: 50 patients were treated with ozone together with surgical and general treatment, and 72 patients were treated with surgical treatment.	Objective: to favor the evolution of purulent wounds in diabetes using ozone therapy. Conclusion: it was possible to observe that ozone did not have a negative impact on homeostasis and the level of intoxication of the body; in addition, they observed a positive effect on the evolution of the wound in purulent processes.
**Navid Faraji. et al. 2021 [18].**	Case report	A 52-year-old patient suffered a traumatic injury to the right tibia due to a fall from a bicycle.	Objective: to study the effectiveness of ozone therapy in the wound of a specific patient. Conclusion: ozone therapy, together with the silver dressing, improves healing, presents antibacterial effects, prevents the appearance of infections, and has a positive effect on glucose and its metabolism.
**Gheuca˘ Solovâstru. et al. 2015 [11].**	Randomized controlled trial	29 patients: 15 patients treated with ozonated oil + a-bisabolol + standard care, and 14 patients treated with standard care + epithelization cream.	Objective: to evaluate the efficacy of an ozonated oil aerosol combined with a-bisabolol in chronic venous ulcers, comparing it with the standard epithelization cream. Conclusion: in this study, it was observed that the ozone in this formula increases its antiseptic, oxygenating, and immunological properties; in addition, a-bisabolol has anti-inflammatory and calming properties, so this formulation offers a promising therapeutic option in the treatment of venous ulcers.
**Yi-Ting Zhou. et al. 2016 [12].**	Randomized clinical trial	92 patients participated: 50 were treated with ozone gas bath + endovenous laser therapy and 42 of them with endovenous laser therapy.	Objective: to demonstrate the effectiveness of the ozone gas bath combined with endovenous laser therapy for the treatment of venous ulcers present in the lower limbs. Conclusion: laser therapy, together with an ozone gas bath, is effective and safe for venous ulcers present in lower limbs; it can be used as an alternative to long-term compression therapy or surgery.
**Faisal Amir. et al. 2022 [13].**	Simple random sampling.	26 patients participated: 13 were treated with bag ozone therapy, and another 13 were treated with standard treatment.	Objective: to investigate the effect of the use of ozone bag therapy in the healing of diabetic foot ulcers. Conclusion: in this study, they observed differences in wound healing in patients with diabetic ulcers, healing better in patients treated with ozone.
**Myroslav V. Rosul. et al. 2016 [15].**	Randomized clinical trial.	47 patients participated, divided into two groups. Group B was treated with traditional therapy, while group A was treated with traditional therapy and systemic ozone therapy, administered for 12–14 days.	Objective: to demonstrate the effectiveness of ozone in complex therapies in patients with diabetic foot. Conclusion: they demonstrated that ozone has a positive effect on the course of the wound process, improving regenerative processes, peroxidation indexes, and the antioxidant system and reducing hospital stays.
